# Supplementary material for: Microarray-based analysis reveals a novel role of the miRNA-613/SNAI2/CXCR4 axis in atrial fibrillation
Source: PLoS One. 2025 Jun 23;20(6):e0324324. doi: 10.1371/journal.pone.0324324 (PMC12184911; doi:10.1371/journal.pone.0324324)
Supplement: Attachment — Submitted filename: Response to reviewer.docx [file pone.0324324.s002.docx]

Point-to-point response

We thank all three reviewers for their insights and suggestions, which have helped us greatly improve the manuscript. Below is a point-to-point response embedded in the reviewer’s comments (in blue).

-----------------------------------------------------------------------------------------------------------------

Response to Reviewers’ comments:

Reviewer(s)' Comments to Author:

Reviewer #1: The study explores key miRNA-mRNA interactions in atrial fibrillation using a bioinformatic approach. While potentially interesting, there are significant shortcomings that affect the interpretability of the results. The methodology was inadequately explained, and there was a lack of reproducible analysis provided. For instance, it is unclear what methods were employed to correct the background and normalize the data. It is also uncertain whether any filters were applied to exclude probes with no expression or if quality control measures such as principal component analysis were conducted. Moreover, confounding corrections do not appear to have been addressed, and it seems that multiple testing corrections (e.g., false discovery rate) were not utilized. In the context of transcriptomics research, this oversight is unacceptable. Furthermore, the lack of independent validation and the small sample sizes of the investigated datasets undermine the validity of their findings.

We sincerely appreciate your thorough review and valuable comments, which have significantly contributed to improving the quality of our manuscript.

We fully acknowledge that our initial submission lacked sufficient detail in describing the data preprocessing, normalization methods, and quality control measures, which could affect the interpretability and reproducibility of our findings. In response to your constructive feedback, we have carefully and thoroughly addressed these issues in the revised manuscript, particularly the methodological concerns you raised.

1. **Methodological details and quality control measures:**

In the Methods section, we have now provided a detailed description of the preprocessing procedures applied to the publicly available miRNA expression dataset (GSE68475) and mRNA expression dataset (GSE31821). Briefly, For the miRNA expression dataset (GSE68475) downloaded from the public GEO database, raw miRNA microarray data were initially processed using GeneSpring GX software. All raw intensity values lower than 1.0 were first adjusted to 1.0 to minimize interference from extremely low-expressed miRNAs. Subsequently, data normalization was performed by referencing the median expression levels of each miRNA across the 11 normal sinus rhythm (NSR) samples, thereby reducing systematic technical biases between arrays. Additionally, miRNAs with maximum raw signal intensities lower than 20 across all samples were filtered out, ensuring the reliability of the subsequent analyses. Furthermore, we performed principal component analysis (PCA) as an additional quality control measure, and the PCA results demonstrated clear separation between AF and NSR samples in two-dimensional principal component space, indicating good data quality and suitability for subsequent differential expression analyses (Figure1 to reviewer1. A). For another mRNA expression dataset (GSE31821) utilized in this study, the raw microarray data (CEL files) were imported into R software and processed using the Robust Multi-array Average (RMA) method, including background correction, quantile normalization, and probe summarization to generate gene-level expression values. PCA was also performed as a quality control measure, and the results clearly showed distinct clustering of AF and control samples, further confirming data quality and supporting the validity of subsequent analyses (Figure1 to reviewer1. B).

**The specific revised *Materials and Methods* content added is as follows:**

2.1 AF-related miRNA expression profiling

An AF-related miRNA microarray dataset (GSE68475) was downloaded from the NCBI GEO database (NCBI, http://www.ncbi.nlm.nih.gov/geo/). This dataset included miRNA expression levels in right atrial appendage samples collected from individuals with AF or normal sinus rhythm (NSR) undergoing open-heart surgery at Oita University Hospital. In total, this dataset included 10 AF patient samples and 11 NSR patient samples. Raw microarray data were preprocessed and analyzed using GeneSpring GX software. Briefly, raw data values below 1.0 were set to 1.0 to minimize potential interference from very low-expression miRNAs. Next, the data for each miRNA were normalized to the median expression level of that miRNA across the 11 NSR samples, eliminating inter-array systematic errors and technical bias. Following normalization, we applied additional filtering to retain only miRNAs with raw signal intensities of ≥20 in at least one sample, ensuring the reliability of subsequent analyses. Principal component analysis (PCA) was performed as a quality control measure, and results demonstrated clear separation between AF and NSR groups, supporting the suitability of the dataset for differential expression analysis.

2.2 Differentially expressed miRNA identification

Differentially expressed miRNAs between AF and NSR patient samples were identified using the R limma package. Briefly, moderated t-tests were performed to compare miRNA expression between the two groups, and P values obtained from these tests were subsequently adjusted for multiple comparisons using the Benjamini-Hochberg False Discovery Rate (FDR) method. MiRNAs with adjusted P-value < 0.05 were considered statistically significant and selected for further analyses.

2.4 Identification of overlapping differentially expressed miRNAs and mRNAs associated with AF

The GSE31821 dataset was downloaded from the GEO database and included auricular tissue biopsy samples from 2 control individuals and 4 AF patients from which RNA were extracted for Affymetrix microarray analysis. Raw microarray data were imported into R and preprocessed using the Robust Multi-array Average (RMA) method, including background correction to reduce background noise, quantile normalization to eliminate technical variability among arrays, and probe summarization to obtain gene-level expression values. Principal component analysis was performed as a quality control measure, revealing clear separation between AF and control samples, supporting data reliability for subsequent analyses.

2. **Multiple testing correction:**

Concerning multiple testing correction, as recommended, we have now implemented the Benjamini-Hochberg False Discovery Rate (FDR) method in our revised analysis. Differentially expressed miRNAs and mRNAs were re-selected based on the adjusted P-value threshold (p-value adj < 0.05), and these updated analytical results have been clearly included in the revised manuscript. Following this adjustment, we identified a slightly reduced number of differentially expressed miRNAs (33 significantly upregulated and 34 downregulated) (Figure2 to reviewer1. A). Importantly, our key miRNAs of interest (including miR-613 and miR-339-5p) remained significantly differentially expressed, demonstrating that our core conclusions are robust to this statistical refinement. Additionally, the set of differentially expressed mRNAs remained unchanged after adjusting the P-values, possibly due to the relatively small sample size limiting the impact of this correction (Figure2 to reviewer1. B).

3. **Adjustment for potential confounding factors and independent experimental validation:**

Regarding your concern about potential confounding factors, we did not perform adjustments for possible confounders such as age and other clinical characteristics, mainly due to the lack of sufficiently detailed metadata available from the public datasets used in this study. We fully understand and sincerely accept your criticism, acknowledging this limitation as an inherent constraint of our study design. We have explicitly clarified this limitation in the revised manuscript and committed to addressing this issue through more rigorous and comprehensive experimental designs in future studies, utilizing datasets with more complete clinical information to minimize potential confounding effects and enhance the robustness of our conclusions.

We fully recognize your concerns regarding the absence of independent validation experiments in our initial submission. To address this important limitation, we have now conducted qPCR validation experiments to independently verify the miRNA-mRNA regulatory relationships predicted by our bioinformatic analysis. Specifically, we performed qPCR experiments to validate the regulatory relationships between miR-613 and its predicted targets SNAI2 and CXCR4, as well as between miR-339-5p and its predicted target FHL1.

Our qPCR results demonstrated clear evidence supporting these interactions: inhibition of miR-339-5p significantly increased FHL1 expression (P<0.0001), whereas the miR-339-5p mimic significantly decreased its expression (P<0.01); inhibition of miR-613 significantly elevated the expression levels of SNAI2 (P<0.0001) and CXCR4 (P<0.01), while the miR-613 mimic significantly reduced their expression (SNAI2, P<0.01; CXCR4, P<0.001) (Figure3 to reviewer1. A-C).

These experimental data strongly validate our computational predictions and substantially enhance the robustness and interpretability of our findings. We have clearly presented these new experimental results in the revised manuscript (Figure 8 in the revised manuscript), and thoroughly discussed the implications in the revised Discussion section.

**The newly added experimental validation section (Results 3.4) in the revised manuscript is:**

3.4 Experimental validation of miRNA-mRNA interactions via quantitative PCR

To experimentally validate the predicted miRNA-mRNA regulatory relationships derived from bioinformatics analyses, quantitative polymerase chain reaction (qPCR) assays were performed. Specifically, we investigated the direct effects of miR-339-5p and miR-613 on the expression levels of their predicted target genes (FHL1, SNAI2, and CXCR4) in vitro (Figure 8).

The qPCR results demonstrated that the expression of FHL1 significantly increased when cells were treated with the hsa-miR-339-5p inhibitor compared to the inhibitor negative control (Inhibitor-NC) group (P < 0.0001), indicating a strong negative regulatory relationship. Conversely, treatment with the miR-339-5p mimic markedly decreased the FHL1 expression levels compared with the mimic negative control (Mimic-NC) group (P < 0.01). These findings clearly support the prediction that miR-339-5p negatively regulates FHL1, reinforcing the complexity of miRNA-mRNA interactions in atrial fibrillation.

Similarly, our analysis confirmed that miR-613 significantly modulated the expression of its putative target genes, SNAI2 and CXCR4. Inhibition of miR-613 led to a substantial upregulation of SNAI2 (P < 0.0001) and CXCR4 (P < 0.01), compared with the Inhibitor-NC group. In contrast, overexpression of miR-613 using a miR-613 mimic significantly decreased the expression of SNAI2 (P < 0.01) and CXCR4 (P < 0.001) compared with the Mimic-NC group. These experimental validations confirm the bioinformatics predictions and substantiate our hypothesis that the downregulation of miR-613 observed in atrial fibrillation patients could contribute to disease progression by relieving repression of SNAI2 and CXCR4 expression.

Collectively, these qPCR validation results provide robust experimental evidence confirming the predicted regulatory interactions and strengthen the mechanistic insights into miRNA involvement in atrial fibrillation pathogenesis.

**The updated limitation section in the Discussion now states:**

Nevertheless, some limitations must also be acknowledged. Due to the lack of detailed clinical metadata in publicly available datasets, adjustments for potential confounding factors such as age and clinical characteristics were not possible, which might affect the robustness of our results. Furthermore, the sample size, particularly for the mRNA dataset, was relatively small, which may limit the generalizability and stability of our conclusions. Although our qPCR validation experiments provided direct evidence supporting the bioinformatics predictions, additional in vivo experiments and validation in clinical tissue samples with larger cohorts are necessary to further elucidate the precise molecular roles of these miRNA-mRNA interactions in AF.

Again, we deeply appreciate your thorough review and insightful suggestions, and we have earnestly and diligently revised the manuscript. We hope our detailed revisions adequately address your concerns and resolve your doubts about the rigor of our data analysis.

Figure1 to reviewer1. Principal component analysis (PCA) of microarray datasets used in this study.

(A) PCA plot of the miRNA expression profiles from dataset GSE68475, showing clear separation between normal sinus rhythm (NSR, blue dots) and atrial fibrillation (AF, red dots) samples. Ellipses represent 90% confidence intervals for each group.

(B) PCA plot of the mRNA expression profiles from dataset GSE31821, demonstrating distinct grouping of AF patient samples (red dots) and control samples (blue dots), confirming suitable data quality for further analyses.

Figure2 to reviewer1. Volcano plots illustrating differential expression analyses for miRNAs and mRNAs.

(A) Volcano plot displaying the differentially expressed miRNAs identified between AF and NSR samples. Red dots indicate significantly upregulated miRNAs, green dots represent significantly downregulated miRNAs, and black dots represent miRNAs without significant changes.

(B) Volcano plot illustrating the differential expression of mRNAs between AF and control samples. Similarly, red and green dots indicate significantly upregulated and downregulated mRNAs, respectively.

Figure3 to reviewer1. Validation of miRNA-mRNA regulatory relationships by qPCR.

qPCR validation of miRNA-mRNA regulatory relationships in atrial fibrillation. Expression level of FHL1 (A), SNAI2 (B) and CXCR4 (C) following transfection with hsa-miR-613 inhibitor or mimic compared to negative controls. Data represent mean ± SD of three independent experiments. **P < 0.01; ***P < 0.001; ****P < 0.0001.

Reviewer #2: This study, while simple in its approach, leads to important and meaningful conclusions.

I would like to clarify why I believe the manuscript is partially technically valid. To strengthen the conclusions, it would be important to validate the findings observed through bioinformatics analysis with additional techniques, such as IHC, among others. Nevertheless, as preliminary data for further studies, the current study is technically valid.

Additionally, the statistical analysis is somewhat rigorous, but I would recommend using adjusted p-values (p-value adj) < 0.05 (or even 0.10) when selecting differentially expressed miRNAs and mRNAs, rather than relying solely on p-values. A p-value of < 0.05 indicates that the adjusted p-value is likely below 0.05, ensuring that the statistical analysis is both rigorous and appropriate. It would be preferable to consistently use either FC (fold-change) or logFC (log fold-change) throughout the manuscript. In Table 1, logFC is provided, while in the text, FC is used with a threshold of >2 (FC>2). To avoid confusion for readers, I suggest using both terms, perhaps in the table, or consistently using only one of the two throughout the manuscript.

I also suggest expanding the discussion to provide more detail, particularly in explaining the significance of the statistically significant Gene Terms (for BP, CC, MF) in relationship of AF, and what they may represent (Figure 2).

Overall, the results are promising, and I believe it is crucial to validate these findings with additional techniques. Lastly, I recommend reviewing the use of abbreviations throughout the manuscript, ensuring that they are defined in brackets the first time they are mentioned, and thereafter avoiding repetition of the full term.I suggest checking English too, but it seems to be quite fluent. I recommend broadening the discussion on your results by comparing them with what has already been observed in AF or similar conditions. If you don't find any comparison articles (I don't think so) explain in great detail your hypothesis also at the level of molecular mechanisms. This would be very useful for those who will do new in vivo studies and using other techniques to validate this study. So it would give an important strength to this study.

We sincerely appreciate for the positive evaluation and encouraging comments regarding our analytical methods and preliminary results. Your recognition provides us great motivation and confidence to further improve the quality of our manuscript.

In response to your valuable suggestions, we have carefully conducted the following revisions:

1. **Independent experimental validation:**

We fully agree with your recommendation regarding additional experimental validation to strengthen our bioinformatics predictions. Accordingly, we performed quantitative real-time PCR (qPCR) experiments to independently verify the miRNA-mRNA interactions predicted by our analyses.

Our qPCR results clearly confirmed these interactions: inhibition of miR-339-5p and miR-613 significantly increased the expression of their respective target genes FHL1 (P<0.0001), SNAI2 (P<0.0001), and CXCR4 (P<0.01), while mimics of miR-339-5p and miR-613 significantly decreased their expression (FHL1, P<0.01; SNAI2, P<0.01; CXCR4, P<0.001). (Figure 1 to reviewer 2).

These results robustly substantiate our bioinformatics predictions, significantly increasing the credibility and reliability of our conclusions. The experimental validation is now clearly presented in the revised manuscript (Figure 8) and thoroughly discussed.

**The newly added experimental validation section (Results 3.4) in the revised manuscript is:**

3.4 Experimental validation of miRNA-mRNA interactions via quantitative PCR

To experimentally validate the predicted miRNA-mRNA regulatory relationships derived from bioinformatics analyses, quantitative polymerase chain reaction (qPCR) assays were performed. Specifically, we investigated the direct effects of miR-339-5p and miR-613 on the expression levels of their predicted target genes (FHL1, SNAI2, and CXCR4) in vitro (Figure 8).

The qPCR results demonstrated that the expression of FHL1 significantly increased when cells were treated with the hsa-miR-339-5p inhibitor compared to the inhibitor negative control (Inhibitor-NC) group (P < 0.0001), indicating a strong negative regulatory relationship. Conversely, treatment with the miR-339-5p mimic markedly decreased the FHL1 expression levels compared with the mimic negative control (Mimic-NC) group (P < 0.01). These findings clearly support the prediction that miR-339-5p negatively regulates FHL1, reinforcing the complexity of miRNA-mRNA interactions in atrial fibrillation.

Similarly, our analysis confirmed that miR-613 significantly modulated the expression of its putative target genes, SNAI2 and CXCR4. Inhibition of miR-613 led to a substantial upregulation of SNAI2 (P < 0.0001) and CXCR4 (P < 0.01), compared with the Inhibitor-NC group. In contrast, overexpression of miR-613 using a miR-613 mimic significantly decreased the expression of SNAI2 (P < 0.01) and CXCR4 (P < 0.001) compared with the Mimic-NC group. These experimental validations confirm the bioinformatics predictions and substantiate our hypothesis that the downregulation of miR-613 observed in atrial fibrillation patients could contribute to disease progression by relieving repression of SNAI2 and CXCR4 expression.

Collectively, these qPCR validation results provide robust experimental evidence confirming the predicted regulatory interactions and strengthen the mechanistic insights into miRNA involvement in atrial fibrillation pathogenesis.

2. **Statistical rigor and adjusted p-values:**

In accordance with your recommendation, we adopted a more rigorous statistical criterion for identifying differentially expressed miRNAs and mRNAs. Specifically, we replaced the previous threshold (P-value < 0.05) with the adjusted P-value < 0.05 using the Benjamini-Hochberg method. Following this adjustment, we identified a slightly reduced number of differentially expressed miRNAs (33 significantly upregulated and 34 downregulated) (Figure 2 to reviewer2. A). Importantly, our key miRNAs of interest (including miR-613 and miR-339-5p) remained significantly differentially expressed, demonstrating that our core conclusions are robust to this statistical refinement. Additionally, the set of differentially expressed mRNAs remained unchanged after adjusting the P-values, possibly due to the relatively small sample size limiting the impact of this correction (Figure 2 to reviewer2. B). Moreover, to further enhance clarity and reproducibility, we have provided detailed descriptions of the preprocessing, normalization, and quality control steps for both miRNA and mRNA datasets in the revised Methods section.

**The specific revised *Materials and Methods* content added is as follows:**

2.1 AF-related miRNA expression profiling

An AF-related miRNA microarray dataset (GSE68475) was downloaded from the NCBI GEO database (NCBI, http://www.ncbi.nlm.nih.gov/geo/). This dataset included miRNA expression levels in right atrial appendage samples collected from individuals with AF or normal sinus rhythm (NSR) undergoing open-heart surgery at Oita University Hospital. In total, this dataset included 10 AF patient samples and 11 NSR patient samples. Raw microarray data were preprocessed and analyzed using GeneSpring GX software. Briefly, raw data values below 1.0 were set to 1.0 to minimize potential interference from very low-expression miRNAs. Next, the data for each miRNA were normalized to the median expression level of that miRNA across the 11 NSR samples, eliminating inter-array systematic errors and technical bias. Following normalization, we applied additional filtering to retain only miRNAs with raw signal intensities of ≥20 in at least one sample, ensuring the reliability of subsequent analyses. Principal component analysis (PCA) was performed as a quality control measure, and results demonstrated clear separation between AF and NSR groups, supporting the suitability of the dataset for differential expression analysis.

2.2 Differentially expressed miRNA identification

Differentially expressed miRNAs between AF and NSR patient samples were identified using the R limma package. Briefly, moderated t-tests were performed to compare miRNA expression between the two groups, and P values obtained from these tests were subsequently adjusted for multiple comparisons using the Benjamini-Hochberg False Discovery Rate (FDR) method. MiRNAs with adjusted P-value < 0.05 were considered statistically significant and selected for further analyses.

2.4 Identification of overlapping differentially expressed miRNAs and mRNAs associated with AF

The GSE31821 dataset was downloaded from the GEO database and included auricular tissue biopsy samples from 2 control individuals and 4 AF patients from which RNA were extracted for Affymetrix microarray analysis. Raw microarray data were imported into R and preprocessed using the Robust Multi-array Average (RMA) method, including background correction to reduce background noise, quantile normalization to eliminate technical variability among arrays, and probe summarization to obtain gene-level expression values. Principal component analysis was performed as a quality control measure, revealing clear separation between AF and control samples, supporting data reliability for subsequent analyses.

3. **Consistency of fold-change notation:**

Regarding the consistency in the usage of FC and logFC terms throughout the manuscript, we fully agree with your recommendation to avoid reader confusion. Therefore, in the revised manuscript, we have uniformly applied a threshold of logFC ≥ 1 to describe differential expression clearly and consistently.

4. **Expansion of Results and Discussion (Gene Terms significance):**

As you suggested, we have expanded the Results section by providing a detailed explanation of Gene Terms (BP, CC, and MF) enriched in our analysis, further clarifying their potential biological relevance to the pathogenesis of AF.

**The specific revised content added is as follows:**

To evaluate the possible biological effects of the identified differentially expressed miRNAs, miRNAs of interest were subjected to GO, KEGG, and transcription factor enrichment analyses with the FunRich tool (v 3.1.3). For the GO analysis, enriched terms were ranked based on enrichment score values (-logP-value) (Figure 2). The top 3 enriched biological process (BP) terms for these miRNAs were signal transduction (24.8%, P>0.05), regulation of nucleobase, nucleoside, nucleotide and nucleic acid metabolism (19.4%, P>0.05), and transport (8.6%, P>0.05) (Figure 2A). Dysregulation of signal transduction pathways is widely recognized as a crucial factor in AF pathogenesis due to their roles in modulating cardiac electrophysiological properties, ion channel activities, and cellular responses. Aberrant nucleotide metabolism and transport processes may influence cellular energy metabolism, transcriptional regulation, and RNA processing, contributing to atrial remodeling and structural alterations frequently observed in AF patients. Regarding cellular components (CC), the top three enriched terms were nucleus (51.8%, P<0.001), cytoplasm (49.3%, P<0.05), and lysosome (17.4%, P<0.05)(Figure 2B). Enrichment of miRNAs targeting genes predominantly located in the nucleus and cytoplasm aligns with their known roles in gene expression regulation, transcriptional control, and signal transduction, which are all relevant to AF pathophysiology. The significant enrichment of lysosomal components suggests potential involvement of autophagy-lysosome pathways in atrial remodeling and fibrosis, mechanisms known to underlie AF development and progression. Additionally, the top three molecular function (MF) terms were transcription factor activity (7.8%, P>0.05), transporter activity (5.6%, P>0.05), and RNA binding (4.2%, P>0.05) (Figure 2C). Altered transcription factor activity and RNA binding processes can directly modulate gene expression programs implicated in AF, such as fibrosis-associated signaling and inflammatory responses. Similarly, transporter activity changes might influence ion channel functions and intracellular signaling, thus directly contributing to AF-associated electrophysiological disturbances. KEGG pathway analysis results were similarly ranked based on enrichment score values (Figure 2D), revealing that these differentially expressed AF-related miRNAs were most closely associated with proteoglycan syndecan-mediated signaling events (38.6%, P<0.001), syndecan-1-mediated signaling events (37.2%, P<0.001), the TRAIL signaling pathway (37.2%, P<0.001), and the sphingosine 1-phosphate (S1P) pathway (36.7%, P<0.001). Syndecan signaling events are known to regulate cellular adhesion, migration, inflammation, and fibrosis—all central elements involved in atrial remodeling processes observed in AF patients. The TRAIL signaling pathway, typically involved in apoptosis and inflammation, may reflect increased apoptotic and inflammatory activities in atrial tissues affected by AF. Additionally, the S1P signaling pathway is crucial in regulating cardiac electrophysiological stability, cellular proliferation, and migration, suggesting its potential role in AF pathology via promoting structural and electrical atrial remodeling. A transcription factor enrichment analysis was also performed for these miRNAs (Figure 3).

5. **Expanded discussion on molecular mechanisms:**

Additionally, as recommended, we have extensively expanded the Discussion section to articulate our hypotheses at the molecular mechanistic level clearly.

**The detailed revised content is provided below:**

Initial analyses for this study utilized the GSE68475 dataset consisting of 11 NSR and 10 AF patient samples, enabling the identification of 70 miRNAs that were differentially expressed between these two groups of which 33 and 34 were respectively upregulated and downregulated in AF patients. To extend these results, differential mRNA expression between 2 AF patients and 4 NSR patients was assessed using a separate publicly available microarray dataset, identifying 56 and 38 mRNAs that were significantly upregulated and downregulated in AF samples. By evaluating the overlap between this latter dataset and the putative targets of the 70 miRNAs identified in the first dataset led to the identification of three overlapping genes (SNAI2, CXCR4, and FHL1). While miR-613 was downregulated in AF samples, the consistent upregulation of its target genes SNAI2 and CXCR4 was also observed. In contrast, both miR-339-5p and its target FHL1 were upregulated in AF samples. At the molecular mechanistic level, we hypothesize that the downregulation of miR-613 observed in AF samples may relieve its inhibitory effect on SNAI2 and CXCR4, thus contributing to the enhanced expression of these genes. SNAI2, a zinc finger transcription factor involved in epithelial-mesenchymal transition, may mediate structural remodeling in atrial tissues by promoting fibrosis and extracellular matrix deposition, both critical factors associated with AF development. Likewise, CXCR4, a chemokine receptor, plays essential roles in inflammatory cell recruitment and tissue remodeling—processes known to contribute significantly to atrial fibrosis and the structural remodeling characteristic of AF pathology. Conversely, the coordinated upregulation of miR-339-5p and its target FHL1 suggests a more complex regulatory mechanism in AF. FHL1 is associated with cytoskeletal dynamics and structural integrity in cardiomyocytes; thus, its upregulation might reflect compensatory or maladaptive remodeling responses occurring during AF progression. These proposed regulatory relationships underline the potential significance of these miRNA-mRNA interactions in the pathogenesis and maintenance of AF. Enrichment analyses of the differentially expressed miRNAs revealed that they were enriched in the signal transduction, regulation of nucleobase, nucleoside, nucleotide, and nucleic acid metabolism, and transport BP terms, the nucleus, cytoplasm, and lysosome CC terms, and the transcription factor activity, transporter activity, and RNA binding MF terms. They were further enriched in the proteoglycan syndecan-mediated signaling events, syndecan-1-mediated signaling events, TRAIL signaling pathway, and S1P pathway KEGG terms. Of particular interest, the TRAIL signaling pathway and S1P signaling pathway have previously been implicated in cardiovascular remodeling processes, inflammation, and apoptotic regulation, suggesting their potential involvement in atrial structural remodeling and electrical instability observed in AF. The syndecan-mediated signaling pathways are also noteworthy, given their known roles in extracellular matrix remodeling and cellular adhesion, both crucial factors influencing cardiac fibrosis and the atrial remodeling processes underlying AF pathogenesis. The most enriched BP terms for the three overlapping target mRNAs were sensory perception of mechanical stimulus, regulation of metal ion transport, regulation of cell growth, and cell growth. Notably, regulation of metal ion transport and cell growth are processes closely related to cellular electrophysiology and myocardial structural remodeling, both of which are critical events in the progression of AF.

6. **Abbreviations and English language check:**

Finally, regarding the appropriate use of abbreviations, we have carefully reviewed and corrected all abbreviations throughout the manuscript. We ensured that each abbreviation was clearly defined upon first use and consistently employed thereafter. Furthermore, we have also thoroughly reviewed and refined the manuscript's English to improve clarity and readability.

We would like to once again express our sincere gratitude for your valuable and constructive suggestions. We have conscientiously addressed each point raised, and believe that these modifications have significantly improved the reliability, clarity, and scientific rigor of our manuscript.

Figure1 to reviewer2. Validation of miRNA-mRNA regulatory relationships by qPCR.

qPCR validation of miRNA-mRNA regulatory relationships in atrial fibrillation. Expression level of FHL1 (A), SNAI2 (B) and CXCR4 (C) following transfection with hsa-miR-613 inhibitor or mimic compared to negative controls. Data represent mean ± SD of three independent experiments. **P < 0.01; ***P < 0.001; ****P < 0.0001.

Figure2 to reviewer2. Volcano plots illustrating differential expression analyses for miRNAs and mRNAs.

(A) Volcano plot displaying the differentially expressed miRNAs identified between AF and NSR samples. Red dots indicate significantly upregulated miRNAs, green dots represent significantly downregulated miRNAs, and black dots represent miRNAs without significant changes.

(B) Volcano plot illustrating the differential expression of mRNAs between AF and control samples. Similarly, red and green dots indicate significantly upregulated and downregulated mRNAs, respectively.
