# Supplementary material for: Microarray-based analysis reveals a novel role of the miRNA-613/SNAI2/CXCR4 axis in atrial fibrillation
Source: PLoS One. 2025 Jun 23;20(6):e0324324. doi: 10.1371/journal.pone.0324324 (PMC12184911; doi:10.1371/journal.pone.0324324)
Supplement: Attachment — Submitted filename: Response to Reviewers.docx [file pone.0324324.s003.docx]

Point-to-point response

We thank all editors and reviewers for their insights and suggestions, which have helped us greatly improve the manuscript. Below is a point-to-point response embedded in the reviewer’s comments (in blue).

-----------------------------------------------------------------------------------------------------------------

Response to Editors’ comments:

Additional Editor Comments:

1) The revised manuscript highlights a new potential adaptive mechanism involving genes regulated by miRNA613 that deserve attention by the authors. Myocardial hibernation refers to a state of reduced contractile function and metabolism in viable human myocardium due to chronic hypoperfusion or dyssynchrony even if the heart is failing (please see J Cell Mol Med. 2014 Mar;18(3):396-414.;J Card Fail. 2009 Dec;15(10):920-8 ). In the setting of AF, particularly when persistent or accompanied by HF, ventricular myocardial hibernation may occur as an adaptive response to chronic hemodynamic stress and altered perfusion. In patients with AF, the downregulation of miRNA-613 leads to upregulation of SNAI2 and CXCR4, which in turn may contribute to fibrotic and structural remodeling (via SNAI2),enhanced cellular survival signaling and adaptation to chronic stress (via CXCR4), possible dedifferentiation of cardiomyocytes, reducing their metabolic activity and contractile function. These changes are consistent with a myocardial hibernation phenotype—where cells downregulate activity to preserve viability under chronic stress. Thus, the miRNA-613/ SNAI2/CXCR4 axis may act as a molecular switch or modulator of hibernation-like states in ventricular myocardium secondary to chronic AF. The authors are invited to include this perspective in their discussion including abovementioned articles.

We sincerely appreciate your thoughtful comments, which have greatly improved the rigor and depth of our manuscript. According to your suggestion, we have expanded the discussion section to include the potential adaptive mechanism involving myocardial hibernation mediated by the miRNA-613/SNAI2/CXCR4 axis, referencing the recommended literature. Specifically, the newly added section is as follows:

Moreover, the downregulation of miRNA-613 and subsequent upregulation of SNAI2 and CXCR4 suggest that ventricular myocardium might enter a state analogous to myocardial hibernation. Myocardial hibernation refers to a protective remodeling process in which cardiac myocytes downregulate their contractile function and metabolic demand to maintain viability under conditions of chronic ischemia or hemodynamic stress. Typical characteristics include reduced myocardial contractility despite preserved cell viability, glycogen accumulation, decreased capillary density, downregulated metabolic enzyme activity, and activation of ischemia-related signaling pathways [62]. It has been reported that even in the absence of overt coronary ischemia, persistent rapid pacing and ventricular dyssynchrony can induce myocardial changes mimicking hibernation, such as increased glycogen storage and reduced expression of glycolytic enzymes, representing an adaptive response to sustained hemodynamic stress [63]. The transcriptional repressor SNAI2 promotes cellular survival under stress conditions, while elevated CXCR4 expression is considered a hallmark of protective myocardial responses under ischemic conditions. Therefore, the observed miRNA-613/SNAI2/CXCR4 axis might function as a molecular modulator facilitating a hibernation-like phenotype in ventricular myocardium under chronic AF-induced hemodynamic stress, ultimately protecting myocardial tissue from energetic depletion and stress-induced damage.

2) Include in the title the miRNA-613/ SNAI2/CXCR4 axis.

We sincerely appreciate for your valuable suggestion. We have revised the title of our manuscript to clearly highlight the miRNA-613/SNAI2/CXCR4 axis, as recommended. The revised title is as follows:

Microarray-based analysis reveals a novel role of the miRNA-613/SNAI2/CXCR4 axis in atrial fibrillation

Response to Reivewers’ comments:

Reviewer #2: I believe that, with the methodological adjustments and revisions made, this study can now be considered valid and suitable for recommendation. The authors have clearly taken my comments into account and have implemented the requested changes appropriately.

Firstly, I appreciated their use of the adjusted p-value, even though some data were modified compared to the previous version. Nevertheless, the key significant results originally highlighted remain evident, thereby further supporting their hypothesis. This consistency demonstrates the robustness of their main conclusions in light of the refined statistical approach.

Moreover, I commend the authors for incorporating qPCR validation, especially as I had suggested using additional techniques to support the bioinformatics findings. This experimental validation has confirmed the observations derived from the bioinformatic analysis, adding substantial value to the study. These results meaningfully reinforce their computational predictions and significantly enhance the credibility and reliability of their conclusions.

Additionally, the revised discussion is more detailed and effectively underscores the authors’ central hypothesis. While in vivo validation would certainly be of interest for future studies, I believe that the analyses presented in this manuscript are sufficient for the current scope of the work.

Thank you very much for your insightful suggestions and positive evaluation of our manuscript. Your detailed and constructive feedback has significantly enhanced the scientific rigor and clarity of our work. In future studies, we plan to further validate these findings through comprehensive *in vivo* experiments to deepen our understanding of the underlying molecular mechanisms of atrial fibrillation.
