# Supplementary material for: Microarray-based analysis reveals a novel role of the miRNA-613/SNAI2/CXCR4 axis in atrial fibrillation
Source: PLoS One. 2025 Jun 23;20(6):e0324324. doi: 10.1371/journal.pone.0324324 (PMC12184911; doi:10.1371/journal.pone.0324324)
Supplement: Attachment — Submitted filename: Response to reviewer_20250514.docx [file pone.0324324.s004.docx]

Point-to-point response

We thank academic editor for insights and suggestions, which have helped us greatly improve the manuscript. Below is a point-to-point response embedded in the editor’s comments (in blue).

-----------------------------------------------------------------------------------------------------------------

Response to Editor’s comments:

Editor(s)' Comments to Author:

ACADEMIC EDITOR: Whilst the authors have included data from qPCR experiments (Figure 8) in the most recent revision, there is no reporting of the qPCR methodology, including the samples used, their origin and the in vitro culture methods to generate them, all of which limit the reproducibility of this study should a reader wish to replicate the findings. As such, the manuscript doesn't currently meet PLOS publication criteria which requires that "Experiments, statistics, and other analyses are performed to a high technical standard and are described in sufficient detail."

The authors are strongly invited to update the reporting of their methodology.

Thank you for your constructive feedback. We apologize for the earlier omission of key methodological details. We have now added a detailed description of our qPCR procedures to the **Materials and Methods** section. Thank you again for your guidance in strengthening the scientific rigor and reproducibility of our manuscript.

The newly added content is as follows:

**2.6 Experimental Model and Cell Culture**

Human ventricular cardiomyocytes (AC16; Sigma-Aldrich, Cat# SCC-AC16) were cultured in DMEM/F-12 (Gibco, Cat# 11320-033) supplemented with 10 % fetal bovine serum (FBS; Gibco, Cat# 10099-141) and 1 % penicillin-streptomycin (Gibco, Cat# 15140-122) at 37 °C in a humidified atmosphere containing 5 % CO₂. Cells were maintained mycoplasma-free, authenticated by short-tandem-repeat profiling, and used between passages 4–10 to ensure phenotypic stability. For all experiments, cells were seeded at 2 × 10⁵ cells well⁻¹ in collagen-I-coated 6-well plates (Corning) and allowed to reach ~70 % confluence before transfection.

**2.7 miRNA Mimic/Inhibitor Transfection**

Gain- and loss-of-function studies were performed with synthetic hsa-miR-613 or hsa-miR-339-5p mimics, inhibitors, and matched negative controls (RiboBio, Guangzhou). Oligonucleotides were transfected at a final concentration of 50 nM using Lipofectamine 3000 (Thermo Fisher Scientific, Cat# L3000-015) according to the manufacturer’s instructions. Briefly, Lipofectamine and oligonucleotides were separately diluted in Opti-MEM (Gibco), combined (1:1 vol/vol), incubated for 15 min at room temperature, and added dropwise to the culture medium. Six hours later, the medium was replaced with fresh growth medium. Cells were harvested 48 h post-transfection for RNA extraction.

**2.8 RNA Isolation and Quantitative Reverse Transcription PCR**

Total RNA was extracted with TRIzol™ Reagent (Thermo Fisher Scientific, Cat# 15596-018) and quantified spectrophotometrically (NanoDrop 2000; Thermo Fisher). RNA integrity was verified by agarose-gel electrophoresis. For mRNA analysis, 500 ng RNA were reverse-transcribed with the PrimeScript™ RT Reagent Kit (Takara, Cat# RR037A) using random hexamers; for miRNA analysis, 200 ng RNA were reverse-transcribed with the Mir-X™ miRNA First Strand Synthesis Kit (Takara, Cat# 638313) employing stem-loop primers. Quantitative PCR was performed on a CFX96 Touch™ Real-Time PCR Detection System (Bio-Rad) with TB Green® Premix Ex Taq™ II (Takara, Cat# RR820A). The 20 µL reaction comprised 10 µL 2× TB Green mix, 0.4 µL of each 10 µM primer, 1 µL cDNA, and 8.2 µL nuclease-free water. Cycling parameters: 95 °C 30 s; 40 cycles of 95 °C 5 s and 60 °C 30 s; followed by melt-curve analysis. Primer sequences for GAPDH, FHL1, CXCR4, SNAI2, and U6 (miRNA reference) are listed in table shown below:

Primer sequences used for qPCR

| Target | Forward (5'→3') | Reverse (5'→3') |
| --- | --- | --- |
| GAPDH | GGAAGCTTGTCATCAATGGAAATC | TGATGACCCTTTTGGCTCCC |
| FHL1 | TGCTGCCTGAAATGCTTTGAC | GCCAGAAGCGGTTCTTATAGTG |
| CXCR4 | GGGCAATGGATTGGTCATCCT | TGCAGCCTGTACTTGTCCG |
| SNAI2 | GAACTGGACACACATACAGTGATT | GGCTGTATGCTCCTGAGCTG |

**2.9 Data Analysis**

Ct values were averaged from three technical replicates per sample. Relative expression was calculated using the 2^-ΔΔCt method with GAPDH (mRNA) or U6 (miRNA) as internal controls and the corresponding negative-control group (Mimic-NC or Inhibitor-NC) as calibrator. Each experiment was repeated independently three times (n = 3). Data are presented as mean ± SD. Statistical significance between two groups was assessed by unpaired two-tailed Student’s t-test in GraphPad Prism 9; p < 0.05 was considered significant.
